# Nebivolol Increases Nitric Oxide Synthase via β_3_ Adrenergic Receptor in Endothelial Cells Following Exposure to Plasma from Preeclamptic Patients

**DOI:** 10.3390/cells11050883

**Published:** 2022-03-04

**Authors:** Thaina Omia Bueno-Pereira, Priscila Rezeck Nunes, Mariana Bertozzi Matheus, Ananda Lini Vieira da Rocha, Valeria Cristina Sandrim

**Affiliations:** Department of Biophysics and Pharmacology, Institute of Biosciences of Botucatu, Universidade Estadual Paulista (UNESP), Distrito Rubiao Junior, Botucatu 18680-000, Sao Paulo, Brazil; thaina.omia@unesp.br (T.O.B.-P.); priscila.nunes@unesp.br (P.R.N.); mariana.bertozzi@unesp.br (M.B.M.); ananda.rocha@unesp.br (A.L.V.d.R.)

**Keywords:** nebivolol, nitric oxide, adrenoceptor, preeclampsia, endothelial dysfunction

## Abstract

Background: Low bioavailability of nitric oxide (NO) is related to the pathophysiology of preeclampsia (PE). In the present study, we investigated the effect of nebivolol (NEB), a β_3_-receptor agonist with vasodilator properties, on the NO synthesis in endothelial cells incubated with plasma from preeclamptic patients. Methods and results: Human umbilical vein endothelial cells (HUVECs) were incubated with plasma from healthy pregnant (HP) and PE women; NO quantification was assessed by a fluorescence compound. We found that endothelial cells incubated with plasma from women with PE show lower NO levels compared with the HP group (*p* < 0.0001). However, NEB treatment increases NO levels, partially, mediated by β_3_ adrenergic receptors (*p* < 0.0001) and through eNOS activation (*p* < 0.0001). Conclusions: Our results suggest that NEB acts in NO synthesis through eNOS activation and β_3_ adrenergic receptors in the endothelium. However, further studies will be needed to understand this molecule.

## 1. Introduction

Nebivolol (NEB) is a third-generation beta-blocker combining highly selectivity for the β_1_ adrenoceptors and the ability to release nitric oxide (NO) through the endothelium [1]. It is a racemic mixture of two enantiomers, D- and L-nebivolol, where L-nebivolol behaves as a β_3_ adrenergic receptor agonist, increasing Ca^+2^ efflux into endothelial cells by activating eNOS and consequently increasing NO bioavailability [2]. Preeclampsia (PE) is associated with a decrease in the bioavailability of NO [3]; the incubation of plasma or serum from women with PE on endothelial cells promotes changes in endothelial cell functions [4].

Therefore, in the present study, we evaluated the effect of NEB addition on NO levels in endothelial cells incubated with plasma from healthy pregnant (HP) women and with PE in the presence of NEB. Additionally, we investigated the molecular mechanisms of NEB in NO synthesis through activation of endothelial nitric oxide synthase and β_3_ adrenoceptors. 

## 2. Methods

The study was approved by the Research Ethics Committee of the Faculty of Medicine of Ribeirão Preto, Brazil (CAAE 37738620.0.0000.5440 approved on 19 October 2020 FMRP–USP) following the principles of the Helsinki Declaration, and all subjects gave written informed consent. Diagnosis criteria of PE were defined by the American College of Obstetricians and Gynecologists [5]. For each pool, 10 plasma samples from healthy pregnant (HP) women and 10 samples from pregnant women with PE were selected.

Human umbilical vein endothelial cells (HUVEC) (EA.hy 926) were cultured until reaching 80–90% of confluence. HUVEC were incubated in supplemented DMEM with 10% (*v*/*v*) plasma from PE or HP and 10 µM of NEB, 100 µM of L-name (NOS inhibitor), 3 µM of L-748,337 (β_3_ antagonist), for 4 h. Cells were used until the 10th passage. 

We used the DAF-FM compound for intracellular NO quantification (5 µM) (Invitrogen, Thermo Fisher Scientific, Carlsbad, CA, USA). The fluorescence signal was measured (excitation 495 nm, emission 535 nm) in a multifunctional plate reader (Synergy 4, BioTek, Winooski, VT, USA). 

Replicates of 5 per group combined with treatments were performed in each experiment. When we compare three or more groups, we used One-Way ANOVA followed by the Holm-Sidak test. Statistical analyses were performed using GraphPad Prism 6.0 (GraphPad Software, San Diego, CA, USA) and for all tests, we considered a *p*-value ≤ 0.05 (two-tailed) significant.

## 3. Results

The general clinical parameters of women in the HP and PE groups whose plasma samples were collected and pooled for the in vitro studies are shown in Table 1.

No differences were identified in the maternal age parameter between the groups. Systolic blood pressure (SBP) and diastolic blood pressure (DBP) is increased in the PE group when compared to the HP group (both *p* < 0.001). Furthermore, the index of body mass (BMI) before pregnancy is also increased in the PE group when compared with the HP group (*p* < 0.001), the gestational age (GA) at delivery, and the weight of the newborn were lower in PE compared to the HP group (*p* < 0.001 and *p* < 0.05, respectively). In addition, no differences were identified in the GA at sampling parameter between the groups. The PE samples were composed of 40% late-onset and 60% early-onset, according to GA at sampling (data not shown).

**Figure 1:**
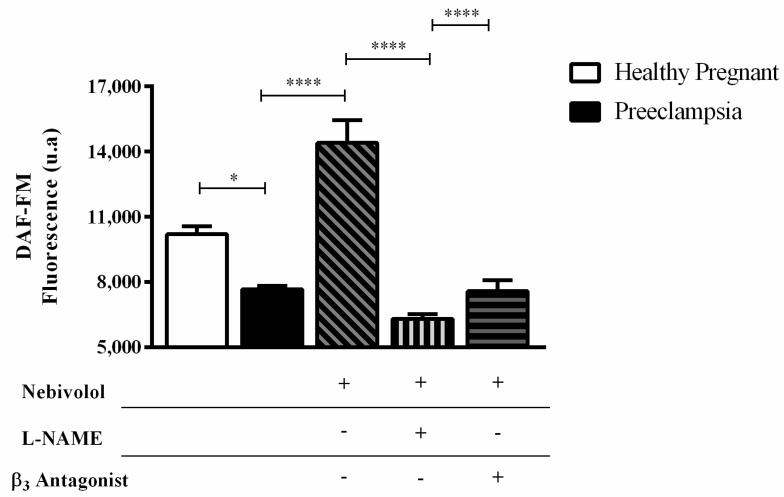
NO fluorescence intensity measured by DAF-FM. HUVECS were incubated with 10% (*v*/*v*) plasma from healthy pregnant (HP, white column) or preeclampsia (PE, black column) (*n* = 10 per group) and treatments for 4 h. Values are means ± S.E.M. Comparisons between groups were realized, assed by One-Way ANOVA followed by Holm-Sidak test. * (*p* < 0.05) HP vs PE; **** (*p* < 0.0001) PE vs PE + Nebivolol; PE + Nebivolol vs PE + L-NAME + Nebivolol; PE + Nebivolol vs PE + β_3_ antagonist + Nebivolol.

Endothelial cells incubated with HP plasma induce higher NO levels when compared with the PE group (*p* < 0.05) (Figure 1). In this group, NEB treatment increased NO levels (*p* < 0.0001) through eNOS activation, as we can identify in the group incubated with L-name (NOS inhibitor) (*p* < 0.0001) (Figure 1). To verify if NEB’s action was mediated by the β_3_ adrenergic receptor it was used the β_3_ antagonist and we found that it reduces NO levels when compared to cultures without antagonist (*p* < 0.0001) (Figure 1). 

## 4. Conclusions

Our results suggest that NEB acts through eNOS activation and β_3_ adrenergic receptors in the endothelial cells following exposure to plasma from preeclamptic patients. The manuscript data lead us to further investigate other mechanisms involving the NO pathway and how nebivolol acts on them in a PE context. We believe that a further understanding of these mechanisms using our strategy model can offer, in the future, alternative management of PE and, consequently, reduce the repercussions of the disease in patients.

## Figures and Tables

**Table 1:** Clinical parameters of HP and PE pregnant women enrolled in the study.

Parameters	HP (*n* = 10)	PE (*n* = 10)
Maternal age (years)	24 ± 1	26 ± 1
SBP (mmHg)	110 ± 3	137 ± 6 *
DBP (mmHg)	71 ± 3	85 ± 4 **
GA at sampling (weeks)	36.25 ± 0.4	32.75 ± 1.7
BMI before pregnancy (Kg/m^2^)	25 ± 1	30 ± 1 **
GA at delivery (weeks)	40 ± 1	32 ± 1 ***
Newborn weight (g)	3175 ± 166	2053 ± 358 *

Values are the means ± S.D. or percentage. HP, Healthy Pregnant; PE, preeclampsia; BMI, body mass index; SBP, systolic blood pressure; DBP, diastolic blood pressure; GA, gestational age. Values are mean ± standard error of the mean. Parametric variables were compared by Student *t*-test and non-parametric by Mann-Whitney test. * *p* < 0.05, ** *p* < 0.01, *** *p* < 0.001 vs healthy pregnant.

## Data Availability

Not applicable.
